# Post‐COVID pulmonary injury in K18‐hACE2 mice shows persistent neutrophils and neutrophil extracellular trap formation

**DOI:** 10.1002/iid3.1343

**Published:** 2024-08-02

**Authors:** Stefanos Giannakopoulos, Juwon Park, Jin Pak, Michelle D. Tallquist, Saguna Verma

**Affiliations:** ^1^ Department of Cell and Molecular Biology, John A. Burns School of Medicine University of Hawai'i at Manoa Honolulu Hawaii USA; ^2^ Department of Tropical Medicine, Medical Microbiology, and Pharmacology, John A. Burns School of Medicine University of Hawai'i at Manoa Honolulu Hawaii USA; ^3^ Center for Cardiovascular Research, John A. Burns School of Medicine University of Hawai'i at Manoa Honolulu Hawaii USA

**Keywords:** K18‐hACE2 mouse model, long COVID, NETs, neutrophils, pulmonary fibrosis, SARS‐CoV‐2

## Abstract

The involvement of neutrophils in the lungs during the recovery phase of coronavirus disease 2019 (COVID‐19) is not well defined mainly due to the limited accessibility of lung tissues from COVID‐19 survivors. The lack of an appropriate small animal model has affected the development of effective therapeutic strategies. We here developed a long COVID mouse model to study changes in neutrophil phenotype and association with lung injury. Our data shows persistent neutrophil recruitment and neutrophil extracellular trap formation in the lungs for up to 30 days post‐infection which correlates with lung fibrosis and inflammation.

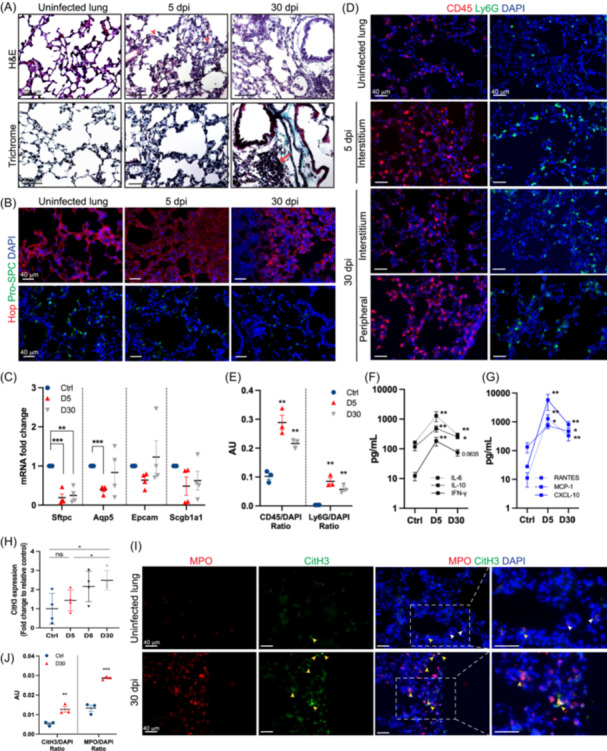

## ETHICS STATEMENT

All experiments were performed on 8–12‐week‐old mice at the dedicated ABSL3 facility according to the animal experimental guidelines issued and approved by the Institutional Animal Care and Use Committee of the University of Hawaii at Manoa.

To the Editor,

Almost 10%–30% of coronavirus disease 2019 (COVID‐19) survivors experience several lingering pulmonary complications, including dyspnea, chronic cough, and pulmonary fibrosis after initial recovery, known as long COVID or postacute sequelae of severe acute respiratory syndrome coronavirus 2 (SARS‐CoV‐2) (PASC).^1^ The pathogenesis of PASC is complex and multifactorial, and underlying mechanisms, specifically, the characterization of immune dysregulation, and whether the persistence of these immune impairments contribute to PASC remain undefined. Neutrophils, the first responders to infection, directly fight pathogens through diverse antimicrobial actions, including the release of their DNA, known as neutrophil extracellular DNA traps (NETs).^2^ However, excessive NETs can also have a detrimental effect on the host by promoting lung inflammation, coagulation cascade, and fibrosis.^3^ Higher serum levels of NET markers (citrullinated Histone H3 [CitH3], cell‐free DNA, and myeloperoxidase [MPO]‐DNA) and NET‐associated granules have been found in severe COVID‐19 patients and associated with disease severity.^4^ NETs were also detected in the microvasculature of lung autopsies from COVID‐19 patients.^5^ Notably, elevated neutrophil numbers and persistent activation status in the circulation have been reported in PASC individuals that correlated with lung function decline.^6^

Collectively, evidence suggests that neutrophil activation is one of the potential drivers of pulmonary complications in severe COVID‐19 patients. However, their involvement in the development of pulmonary sequelae beyond the acute phase is not yet characterized mainly due to the limited accessibility to human bronchoalveolar lavage fluid samples and lung tissues from COVID‐19 survivors. To circumvent this limitation, relevant SARS‐CoV‐2 animal models are needed. So far, no mouse model has been established to characterize long‐term changes in the neutrophil population in the lungs after virus clearance. Both, the K18‐hACE2 mouse model and mouse‐adapted SARS‐CoV‐2 strain (MA‐10) are routinely used to study different aspects of COVID‐19 pathogenesis.^7^, ^8^ The MA‐10 infection leads to milder symptoms with no changes in lung neutrophil counts that does not adequately recapitulate the clinical features seen in PASC. We recently showed that 15%–20% of hACE2 mice survived SARS‐CoV‐2 infection and demonstrated sustained lung inflammation for up to 30 days postinfection (dpi).^9^ In this study, we provide evidence that neutrophil dysregulation is sustained in the postacute stage of K18‐hACE2 mice and associated with chronic pulmonary injury and inflammation.

**Figure 1:**
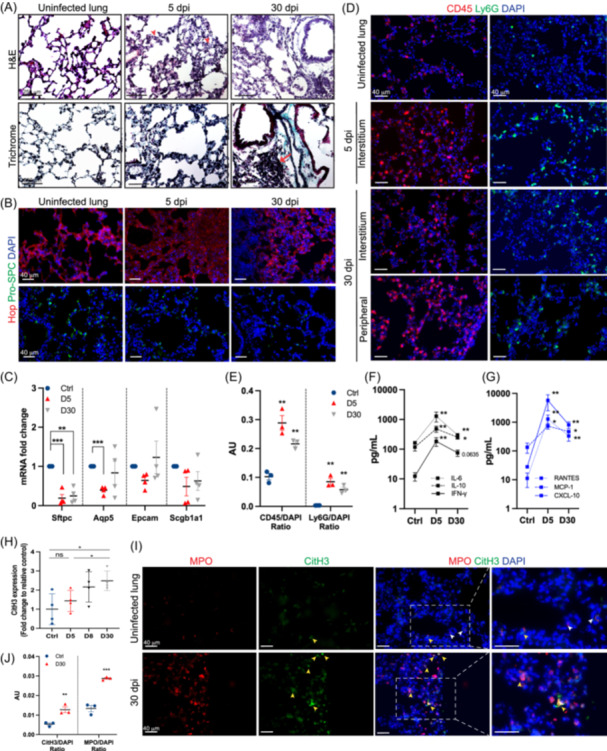
K18‐hACE2 mice were infected intranasally with 10^4^ PFU of SARS‐CoV‐2 (USA‐HI‐B.1.429, similar to Delta variant), sacrificed at 5‐, and 30‐days postinfection (dpi) and perfused lungs were fixed overnight in 4% paraformaldehyde for the pathological and immunological evaluation or flash frozen for RNA and protein analyses. (A) The lung sections (10 μm thickness) stained with hematoxylin and eosin (H&E (top) and trichrome (bottom) showed evidence of immune cell infiltration and fibrosis at 5 and 30 dpi. The red arrowhead and arrow indicate immune cells and iBALT, respectively. *n* = 4 mice per time point. (B and C) Lungs at 30 dpi display sustained alveolar injury. (B) Representative images of the lung sections stained with antibodies against Hop (red) and Pro‐SPC (green) and imaged by epifluorescence microscopy. Magnification 20x; scale bar, 250 μm (*n* = 4 mice per time point). (C) mRNA expression of Epcam, Aqp5, Sftpc, and Scgb1a1 was determined in the lungs using RT‐PCR. Data was normalized using GAPDH and fold change was calculated compared to control lungs. (*n* = 4 mice per time point). (D and E) Leukocyte and neutrophil infiltration is sustained till 30 dpi. (D) Representative images of lung sections stained with antibodies against CD45 (red) and Ly6G (green) at indicated time points and imaged using epifluorescence microscope. Maginfication 20x; scale bar, 40 μm. (E) CD45^+^ or Ly6G^+^ cells were normalized to respective DAPI^+^ cells, quantified using the ImageJ‐particle analyzer tool, and expressed as arbitrary units (AU, *n* = 3 mice per time point). (F and G) The levels of indicated cytokine and chemokines in lung homogenates were measured using Luminex assay. (H and I) An increase in NETs was detected in the infected lungs. (H) Lung lysates prepared in tissue protein extraction reagent with protease and phosphatase inhibitors were used to measure CitH3 protein levels using ELISA. The relative fold change in the infected lungs at 5, 8, and 30 dpi was calculated compared to control lungs (*n* = 4 mice per time point). (I) Representative images showing the presence of NETs in the lungs at 30 dpi. Lung sections were stained for nuclei (DAPI, blue), MPO (red), and CitH3 (green), and imaged by epifluorescence microscope. Magification 20x; scale bar, 40 μm. Colocalization of MPO and CitH3 (orange) in the merged panel is indicated by yellow arrowheads and MPO+ cell is indicated by white arrowheads (*n* = 4 mice per time point). (J) Quantification of CitH3 and MPO in the control and infected lungs at 30 dpi using ImageJ (*n* = 3 mice). Error bars represent ± standard error mean (SEM). Significance was determined using Student's *t*‐test (C, E, H, and J) and Mann–Whitney test (F, G) (*p < .05, **p < .01, ***p < .001). CitH3, citrullinated Histone H3; DAPI, 4′,6‐diamidino‐2‐phenylindole; ELISA, enzyme‐linked immunosorbent assay; H&E, hematoxylin and eosin; NETs, neutrophil extracellular DNA traps; RT‐PCR, real‐time polymerase chain reaction; SARS‐CoV‐2, severe acute respiratory syndrome coronavirus 2.

To better understand neutrophils' involvement in persistent lung injury including fibrosis seen in long‐term COVID patients, the K18‐hACE2 mice were infected with 10^4^ plaque‐forming units of SARS‐CoV‐2 (USA‐HI‐B.1.429, similar to Delta variant) as shown in our previous study.^7^, ^9^ The survivor mice were sacrificed at 30 dpi and perfused lungs were either flash frozen or fixed in 4% paraformaldehyde for the pathological and immunological evaluation and compared with 5 dpi lungs. As expected, hematoxylin and eosin staining of 5 dpi lung sections showed interstitial thickening and immune cell infiltration in the parenchyma (Figure 1A). However, airway wall thickening persisted up to 30 dpi and appeared thicker, compared with 5 dpi lungs. Inflammatory cell infiltrates were observed at both 5 and 30 dpi lungs, but ectopic inducible bronchus‐associated tissues (iBALT) formation was only seen at 30 dpi. Further assessment of fibrotic degree by trichrome staining revealed intense and widely distributed collagen deposition in the lung interstitium at both 5 and 30 dpi lungs while peribronchiolar thickening was observed only at 30 dpi (Figure 1A).

To specifically assess the extent of alveolar epithelial repair after the virus clearance, lung sections were stained for alveolar type I (AT1; Hop) and alveolar type II (AT2; Pro‐SPC) markers. Compared to uninfected controls, Hop expression appeared diffuse and speckled throughout the lungs at 5 dpi. Also, the numbers of Pro‐SPC^+^ cells were markedly reduced (Figure 1B), which is indicative of alveolar epithelial damage followed by the initial infection. Interestingly, on 30 dpi, AT1 cells partially regained Hop expression, and its expression was uneven throughout the lungs. However, the numbers of pro‐SPC^+^ cells were still lower with weak staining intensity, compared to controls (Figure 1B). To take this regional difference into account, we performed quantitative qRT‐PCR analysis of selected lung epithelial cell markers (Epcam; epithelial cell, Aqp5; AT1, Sftpc; AT2, and Scgb1a1; club cell). As expected, expression of all these markers was either significantly lower or tended to be decreased at 5 dpi (Figure 1C). Interestingly, Sftpc expression remained significantly low even at 30 dpi, but other markers partially returned to the baseline expression levels at 30 dpi (Figure 1C).

We then sought to determine if immune cell recruitment is also sustained within the lung after the virus clearance. The presence of CD45^+^ cells (leukocytes) and Ly6G^+^ cells (neutrophils) significantly increased in the alveolar septa and interstitium at 5 dpi and remained higher at 30 dpi than controls (Figure 1D,E). The presence of increased infiltrating immune cells at 30 dpi was also accompanied by sustained levels of key cytokines (IL‐6, IL‐10, and IFNγ) and chemokines (RANTES, MCP‐1, and CXCL10) associated with the migration and activation of immune cells (Figure 1F,G). Therefore, we next assessed if infiltrating neutrophils in the lungs are more likely to form NETs. The levels of CitH3 in the lung homogenates measured using ELISA tended to be higher during the acute stage of the disease, compared to uninfected lungs although it was not statistically significant. However, CitH3 levels at 30 dpi lungs increased significantly by 2.5 ± 0.5 fold compared to uninfected lungs (Figure 1H). This trend was also seen in the immunostaining of NET markers. The NET‐forming cells identified as double positive for CitH3 and MPO (CitH3^+^MPO^+^ cells) were not observed in the control lungs, but they were evident in the lungs at 30 dpi as shown in Figure 1I,J. Thus, collective data suggest the persistence of inflammatory mediators in the lung may lead to neutrophil recruitment and the continued presence of NET during the lung repair/remodeling stage of recovery.

This timely study provides the first evidence that survivor K18 hACE2 mice can be an appropriate small animal model to study the involvement of neutrophils in post‐COVID‐19 pulmonary injury as they more accurately mimic moderate to severe disease in humans who are also more prone to PASC. In comparison, other mouse models including MA10 strain‐infected wild‐type mice representing mild‐moderate disease^10^ may provide insights into pulmonary complications observed in mild COVID‐19. The alterations in circulating neutrophil functions, including upregulation of neutrophil‐associated inflammatory signatures, have already been shown in PASC individuals,^6^ but direct in vivo evidence of neutrophil recruitment and NETs present within the lung after the virus clearance was lacking. Our findings advance the field in several ways by showing (i) sustained lung inflammation and injury (ii) long‐term neutrophil recruitment, and (iii) increased NET formation in the lungs for up to 30 dpi. NETs can contribute to lung injury by potentiating inflammation via the cGAS‐STING pathway, inflammasome activation, and/or interaction with fibrinogen and platelets to cause thrombosis.^11^ Therefore, we speculate that higher levels of inflammatory cytokines lead to sustained neutrophil influx within the lung, affecting immune cell crosstalk that further contributes to the inflammation and delay in alveolar repair post‐recovery in these mice. However, the role of neutrophils‐derived reactive oxygen species and granules in post‐COVID19 pulmonary injury cannot be ruled out and warrants further investigation.

Although circulating NETs are detected in COVID‐19 patients, there are some inconsistencies regarding their association with clinical outcomes. While de Diego and colleagues reported that only cell‐free DNA in the plasma but not other NET markers correlated with disease severity,^12^ other studies have shown the association of multiple markers of NET with severe COVID‐19.^4^, ^13^, ^14^ Our recent study also indicates that mature neutrophils with heightened activation phenotype remained in circulation for up to 6 months in PASC patients with persistent pulmonary sequelae.^15^ Therefore, it is essential to identify clinically relevant NET markers that are highly associated with post‐COVID pulmonary fibrosis and test them as therapeutic targets. Major gaps in our understanding of the involvement of other immune cell types and neutrophil‐specific downstream pathways limit our efforts to ultimately design effective therapeutic strategies to suppress chronic inflammation and/or facilitate alveolar epithelial regeneration. Our preliminary findings open up opportunities to use the K18‐hACE mouse model to further systemically dissect the role of neutrophils and NETs in post‐COVID‐19 pulmonary injury. Importantly, the data provides a framework for future translational research including evaluation of the inhibitors targeting NET or neutrophil activation in preventing PASC development.

## AUTHOR CONTRIBUTIONS

**Stefanos Giannakopoulos**: Conceptualization; data curation; formal analysis; methodology; writing—original draft. **Juwon Park**: Conceptualization; data curation; formal analysis; funding acquisition; investigation; methodology; project administration; writing—original draft. **Jin Pak**: Data curation; investigation; methodology; writing—review & editing. **Michelle D Tallquist**: Funding acquisition; resources; writing—review & editing. **Saguna Verma**: Conceptualization; data curation; formal analysis; funding acquisition; investigation; methodology; supervision; writing—original draft.

## CONFLICT OF INTEREST STATEMENT

The authors declare no conflict of interest.

## Data Availability

All data supporting the findings of this work are available within the paper. The raw datasets generated and analyzed during the current study are available from the corresponding authors upon request.
